# Instability of Misoprostol Tablets Stored Outside the Blister: A Potential Serious Concern for Clinical Outcome in Medical Abortion

**DOI:** 10.1371/journal.pone.0112401

**Published:** 2014-12-15

**Authors:** Veronique Berard, Christian Fiala, Sharon Cameron, Teresa Bombas, Mirella Parachini, Kristina Gemzell-Danielsson

**Affiliations:** 1 ICB - CNRS, Division MaNaPI, Département Nanosciences, Université de Bourgogne, Dijon, France; 2 Gynmed Clinic, Vienna, Austria; 3 Chalmers Centre, NHS Lothian, Edinburgh, Scotland; 4 Obstetric Service, Centro Hospitalar e Universitário de Coimbra, Coimbra, Portugal; 5 San Filippo Neri Hospital, Rome, Italy; 6 Department of Women’s and Children’s Health, Division of Obstetrics and Gynecology, Karolinska Institute and Karolinska University Hospital, Stockholm, Sweden; Baylor College of Medicine, United States of America

## Abstract

**Introduction:**

Misoprostol (Cytotec) is recognised to be effective for many gynaecological indications including termination of pregnancy, management of miscarriage and postpartum haemorrhage. Although not licensed for such indications, it has been used for these purposes by millions of women throughout the world. Misoprostol tablets are most often packaged as multiple tablets within an aluminium strip, each within an individual alveolus. When an alveolus is opened, tablets will be exposed to atmospheric conditions.

**Objective:**

To compare the pharmaco technical characteristics (weight, friability), water content, misoprostol content and decomposition product content (type A misoprostol, type B misoprostol and 8-epi misoprostol) of misoprostol tablets Cytotec (Pfizer) exposed to air for periods of 1 hour to 720 hours (30 days), to those of identical non exposed tablets.

**Methods:**

Four hundred and twenty (420) tablets of Cytotec (Pfizer) were removed from their alveoli blister and stored at 25°C/60% relative humidity. Water content, and misoprostol degradation products were assayed in tablets exposed from 1 to 720 hours (30 days). Comparison was made with control tablets (N = 60) from the same batch stored in non-damaged blisters. Statistical analyses were carried out using Fisher’s exact test for small sample sizes.

**Results:**

By 48 hours, exposed tablets demonstrated increased weight (+4.5%), friability (+1 300%), and water content (+80%) compared to controls. Exposed tablets also exhibited a decrease in Cytotec active ingredient dosage (−5.1% after 48 hours) and an increase in the inactive degradation products (+25% for type B, +50% for type A and +11% for 8-epi misoprostol after 48 hours) compared to controls.

**Conclusion:**

Exposure of Cytotec tablets to ‘typical’ European levels of air and humidity results in significant time-dependent changes in physical and biological composition that could impact adversely upon clinical efficacy. Health professionals should be made aware of the degradation of misoprostol with inappropriate storage of misoprostol tablets.

## Introduction

**Figure 1:**
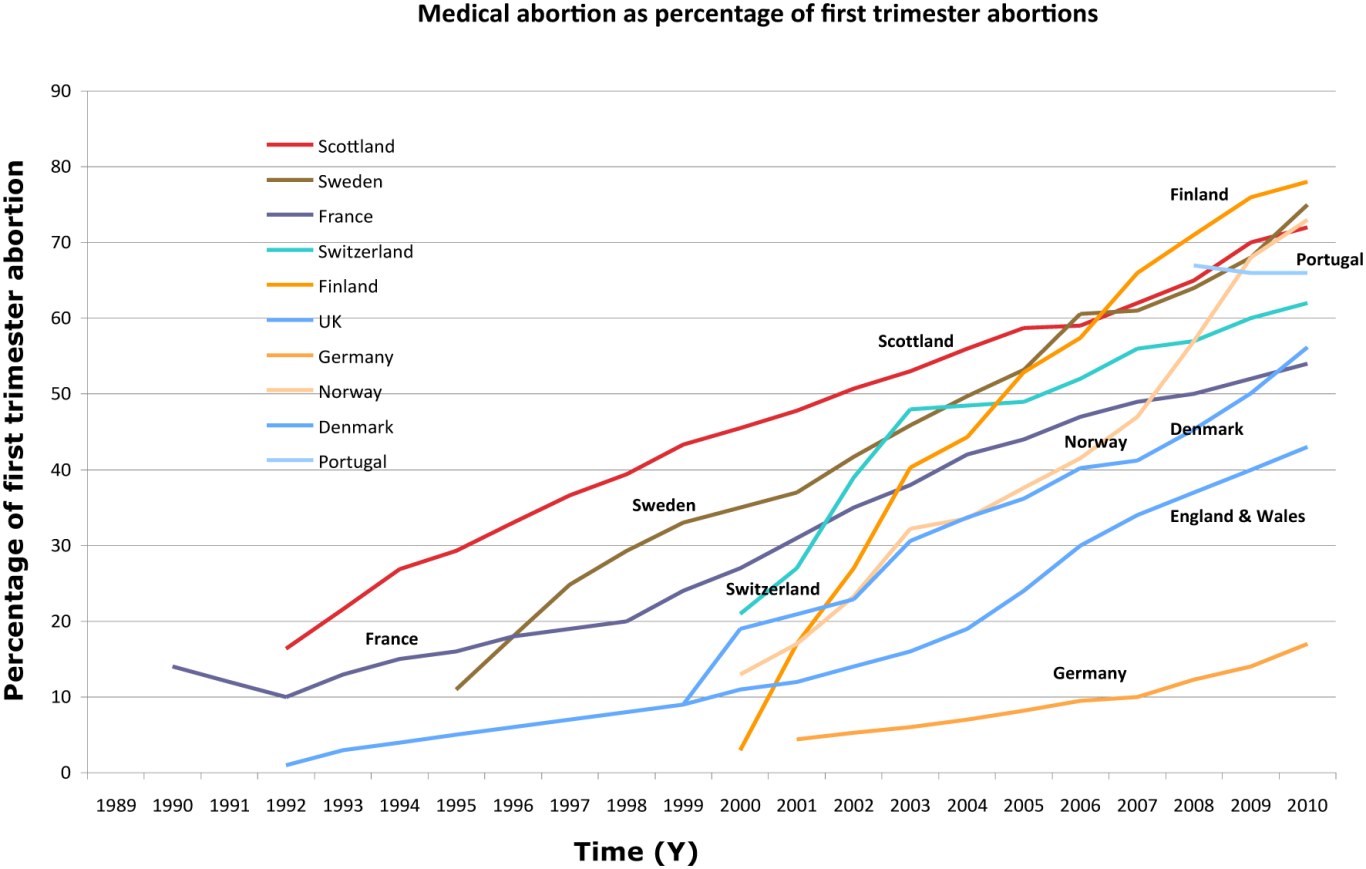
Change over time in medical abortion as percentage of first trimester abortions. Note below the figure: references regarding figures may be found at the following addresses. Scotland: http://www.isdscotlandarchive.scot.nhs.uk/isd/6207.html; Sweden: http://www.socialstyrelsen.se/statistik/statistikefteramne/aborter; France: http://www.drees.sante.gouv.fr/les-interruptions-volontaires-de-grossesse-en-2011,11149.html; Switzerland: http://www.bfs.admin.ch/bfs/portal/de/index/themen/14/02/03/key/03.html; Finland: www.stakes.fi/; UK: https://www.gov.uk/government/collections/abortion-statistics-for-england-and-wales; Germany: https://www.destatis.de/DE/ZahlenFakten/GesellschaftStaat/Gesundheit/Schwangerschaftsabbrueche/Schwangerschaftsabbrueche.html; Norway: http://www.ssb.no/en/abort; Denmark: http://www.dst.dk/da/; Portugal: http://www.spdc.pt/files/Relatorio_Interrupo_de_gravidez_2012.pdf.

Misoprostol (brand name Cytotec) has been approved by European Heath Authorities and by the United States Food and Drug Administration (FDA) for the prevention and treatment of gastric ulcers only. However, clinicians routinely used this misoprostol off-label for obstetric and gynaecological purposes, including cervical ripening, labour induction, and mid-trimester terminations of pregnancy. [1], [2] Multiple clinical studies of misoprostol in this non-approved field have been published. [3], [4], [5], [6], [7] The main goal of these studies was to establish the lowest effective dose in order to keep the rate of side effects as low as possible. As a result of this research effort, misoprostol is now widely recommended for these clinical indications throughout the world. [8], [9], [10], [11] Misoprostol is increasingly used in combination with mifepristone for the termination of early pregnancies. (Fig. 1) [12], [13], [14], [15] Despite recent introduction of other misoprostol preparations on European market, Cytotec is still by far the preparation that is the most currently used. The packaging of Cytotec consists of multiple tablets in an hermetic aluminium strip, each within a blister. Depending on clinical practice in different clinical settings, there may be quite varied modes of delivery and storage of misoprostol tablets.

In clinical settings (such as Sweden, France, Austria, Portugal and USA) where women requesting an early medical termination of pregnancy are able to choose to take misoprostol at home, healthcare professionals commonly deliver 2 or more tablets of 200 µg misoprostol to the woman at the initial visit (when she receives mifepristone) with instructions for her to take the misoprostol tablets at home 48 h later. [16] Under such circumstances it is quite possible that a blister strip is cut during this first visit and the other part of the blister is kept in a desk drawer in the office or pharmacy cupboard until the next request. It is also possible, in busy clinical settings such as hospitals, that blisters may be pre-cut in advance, to facilitate the conduct of medical procedures. Unfortunately the arrangement of misoprostol tablets within the packaging is such that it can be technically difficult to cut tablets from a blister without inadvertently damaging/opening one or more alveoli.

It is generally considered that Cytotec within a blister is stable at room temperature and this has often been cited as an advantage of use of this preparation over vaginal pessaries such as Cervagem, that require refrigeration. [17] However, there is evidence that misoprostol (as for all prostaglandin E1 in general) is chemically unstable at room temperature outside of the packaging. [18] This is due to both relative humidity (RH) and temperature factors. In the unfavourable storage conditions represented by opened alveoli, misoprostol turns into 3 main inactive degradation products: type A and type B and 8-epimer misoprostol [18]. The inactive type A misoprostol is obtained by dehydration, and 8-epi misoprostol by isomerization, which are both catalysed by water; type B misoprostol is the result of isomerisation of inactive type A [19].

The aim of this research was to study the changes in the stability of misoprostol (Cytotec, Pfizer) associated with exposure of the tablet to typical European air/humidity conditions, such as might be the case with inadvertent opening of the alveoli. This is important since changes in biological activity of the tablets could impact negatively upon their clinical efficacy. Specifically the objective of this study was to evaluate changes in the tablets when stored outside their blister pack (worst case) in conditions of humidity and temperature equivalent to weather conditions in European area, from a few hours up to 1 month.

## Materials and Methods

For this study, 8 commercial boxes of 60 units of 200 µg Cytotec were used, corresponding to one box for each duration of exposure. Four hundred and twenty (420) tablets of Cytotec (Pfizer), i.e. 7 boxes, from the same batch (n°B00460) were taken out of their blister and stored in a climatic chamber (WTB BINDER GmbH) at 25°C/60% RH, considered as median usual European conditions to be used for stability studies according to the European Medical Agency, [20] during respectively 1 hour (T1h), 6 hours (T6h), 1 day (T24h), 2 days (T48h), 7 days (T168h), 15 days (T360h) and 30 days (T720h). The reference was represented by the 60 Cytotec tablets (1 box) stored in non damaged blisters, named T0. The number of tablets used for the study was the minimum number of tablets necessary to conduct studies in accordance with Pharmacopeia standards. [21], [22], [23], [24], [25] For each parameter, the analysis was performed on a sample of tablets at each time point.

For tablets water content determination, because the method for determining the solvent content uses aqueous solvant, the gas chromatography method could not be used. Instead, analyses were performed using Karl Fischer direct titration of water (Karl Fisher Metrohm: 787 KF Titrino), according to European Pharmacopeia 2.5.12. Method A, [21] with a sample amount of 150 mg of 10 finely ground tablets.

Simultaneously the pharmaco-technical characteristics of Cytotec tablets were studied according to the European pharmacopeia i.e.: Tablet mass uniformity was analysed using 20 individually weighted tablets (METTLER TOLEDO AE240). [22] The friability test was to measure the total mass of 10 tablets placed in a drum before and after 100 rotations. The percentage loss in mass gives the tablet friability (PHARMA TEST PTF E). [23] The tablets diametric tensile strength was analysed on 10 tablets, with measurement of the force, expressed in Newton, required to tablet crushing (PHARMA TEST PTB 511). [24] The dimensions of tablets; i.e. diameter and thickness, were measured using 10 tablets (ABSOLUTE DIGIMATIC MITUTOYO).

Cytotec tablets were also analysed to determine their misoprostol and decomposition products dosage as follows: type A misoprostol (Pharm. Eur. impurity C), type B misoprostol (Pharm. Eur. Impurity D) and 8-epi misoprostol (Pharm. Eur. impurity A). High-performance liquid chromatography (HPLC) was found to be the best method for both performing the assay and determining the level of the degradation products (HPLC LACHROM ELITE VWR 13) according to European Pharmacopeia monography of misoprostol. [25] Each sample was made of ten finely ground tablets The mobile phase preparation was a mix of 480 mL of water with 520 mL of acetonitrile, the wavelength was 200 nm with a flow rate of 1 ml/min and an expected retention time of 8.5 minutes. All HPLC tests used for determining misoprostol or decomposition products dosage were carried out in presence of corresponding standards.

Tablets water content, pharmaco-technical characteristics- i.e. weight, thickness and diameter- and composition in misoprostol and decomposition products were compared between the samples of tablets exposed to environmental conditions for different durations and reference tablets (T0). Any change above 10% for tablets characteristics, i.e; diameter, thickness, resistance, and weight leads to data outside the range described by the European Pharmacopeia and therefore to non-conform tablets [22], [23], [24].

Statistical analyses were carried out using Fisher’s exact test for small sample sizes. Statistical significance was assigned to p-values<0.05.

## Results

Values for each measured parameter at T0 and T48h are provided in Table 1.

**Table 1:** Changes in misoprostol tablet characteristics and content in misoprostol and misoprostol degradation products after 24 and 48 h exposition to usual European humidity and room temperature.

		Time	
	T0	T24	T48
	N = 60	N = 60	N = 60
**Tablets characteristics**			
Tablets water content (mg/tablets); mean	6,60	11,00	11,80
95% CI	6,49–6,71	10,82–11,18	11,60–11,99
Tablets water content (%); mean	3,28	5,53	5,93
95% CI	3,09–3,47	5,48–5,58	5,85–6,00
Weight (mg); mean	200,04	206,91	208,59
95% CI	199,18–200,89	206,17–207,64	207,84–209,35
Thickness average (mm); mean	3,50	3,63	3,67
95% CI	3,48–3,52	3,62–3,65	3,62–3,71
Diameter (mm); mean	8,400	8,477	8,502
95% CI	8,397–8,403	8,471–8,489	8,498–8,506
Hardness (N); mean	139,55	99,32	94,86
95% CI	134,22–144,88	95,83–102,91	89,12–100,60
Friability average (%); mean	0,036	0,44	0,51
95% CI	0,027–0,044	0,36–0,54	0,45–0,58
**Tablet content in misoprostol and misoprostol degradation products**			
Misoprostol (µg/tablets); mean	215,99	210,01	204,93
95% CI	215,16–216,82	207,57–203,45	202,21–207,65
A-type misoprostol (g/100 g misoprostol); mean	0,31	0,33	0,45
95% CI	NA	NA	NA
B-type misoprostol (g/100 g misoprostol); mean	0,03	0,04	0,04
95% CI	NA	NA	NA
8-épi misoprostol (g/100 g misoprostol); mean	0,09	0,10	0,10
95% CI	NA	NA	NA

CI: confidence interval; NA: not available; *: p<0.05 vs T0.

### Tablets water content results

**Figure 2:**
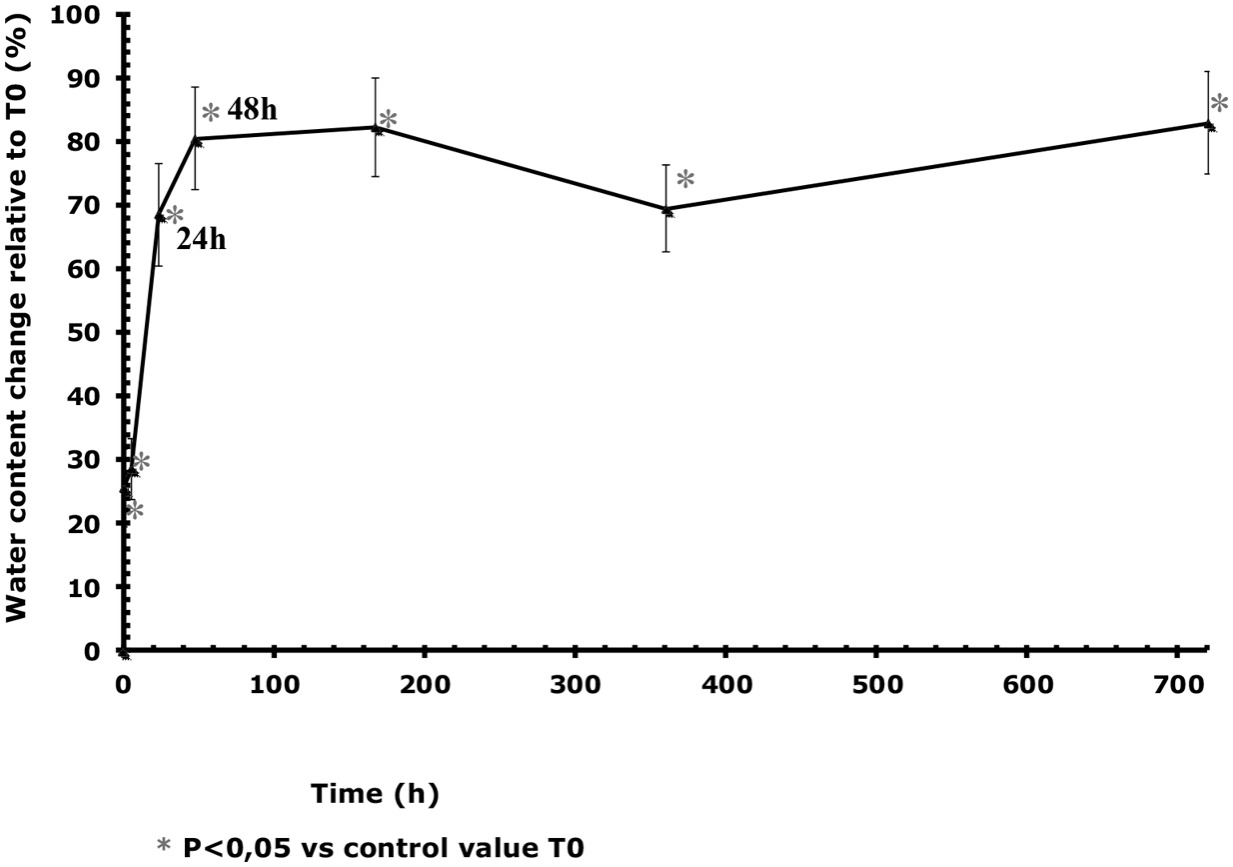
Changes over time in percentage of water content of tablets stored at 25°C/60% RH in comparison with to T0. The vertical bars represent the 95% confidence interval limits for the measured water levels.

Under environmental conditions studied, the water content of tablets significantly increased by 80% in 2 days in comparison with tablets stored in their blister. After 24 hours, the tablet water content reached 5.53% (Table 1). The changes in water content of tablets relative to T0 over time are presented in Fig. 2.

### Pharmaco-technical characteristics of Cytotec tablets

**Figure 3:**
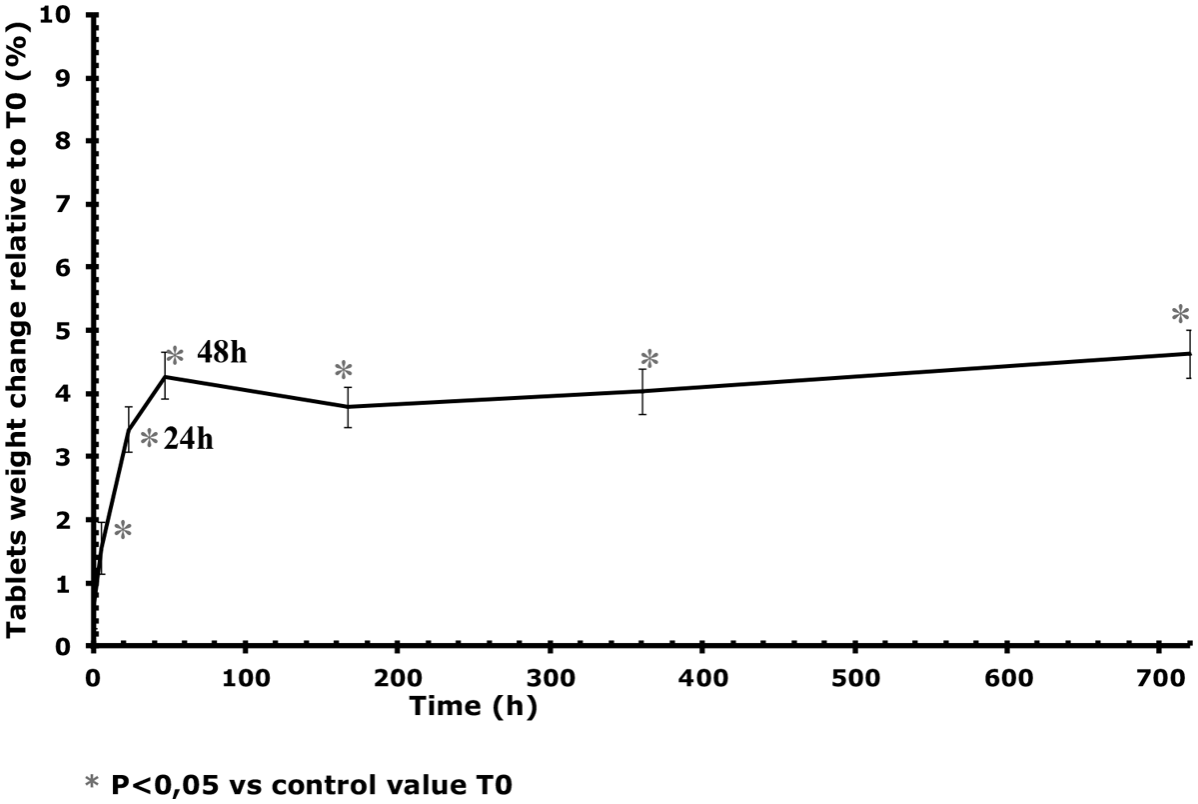
Changes over time in the weight of tablets stored at 25°C/60% RH in comparison with T0. The vertical bars represent the 95% confidence interval limits for the measured weight variation.

**Figure 4:**
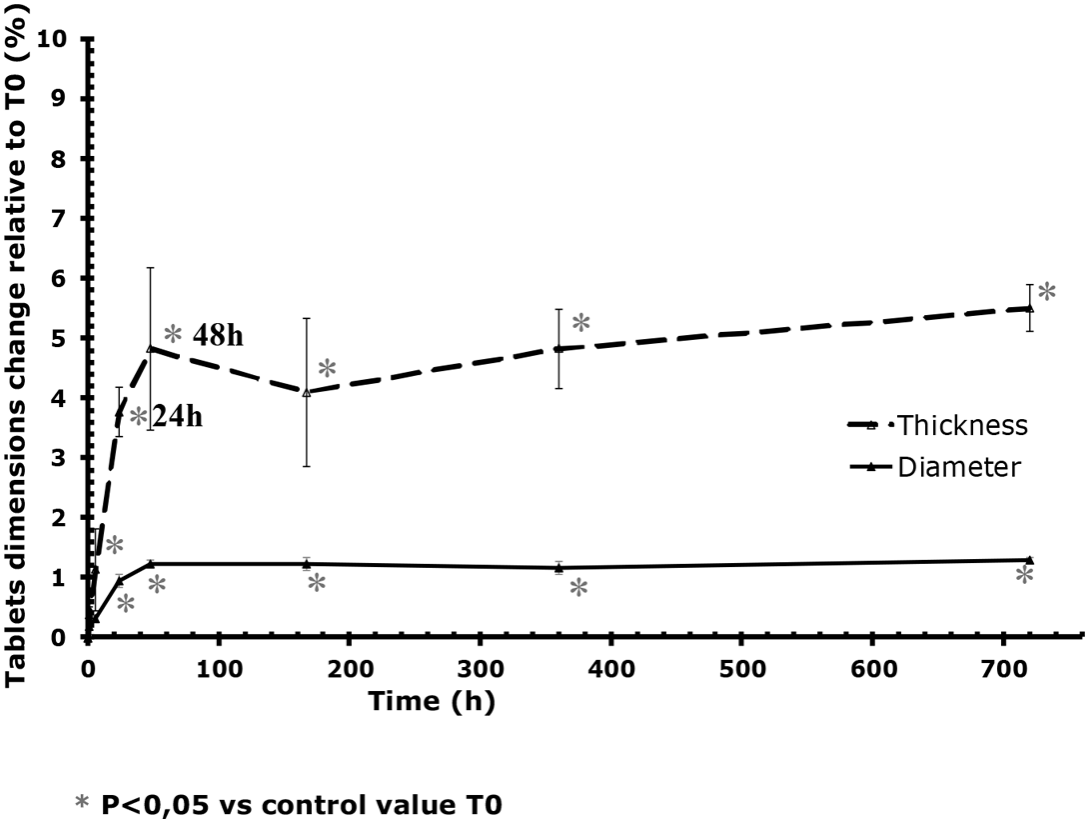
Changes over time in the diameter and thickness of tablets stored at 25°C/60% RH in comparison with T0 over time. The vertical bars represent the 95% confidence interval limits for respectively the measured diameter variation and the measured thickness variation.

In contact with atmospheric water, after 48 hours (maximum effect time), there was a statistically significant 4.5% increase in the weight of tablets in comparison with the reference tablets, and an increase of 1.2% in tablets’ diameter and of 5% in tablets’ thickness (Table 1, Figs. 3 and 4).

**Figure 5:**
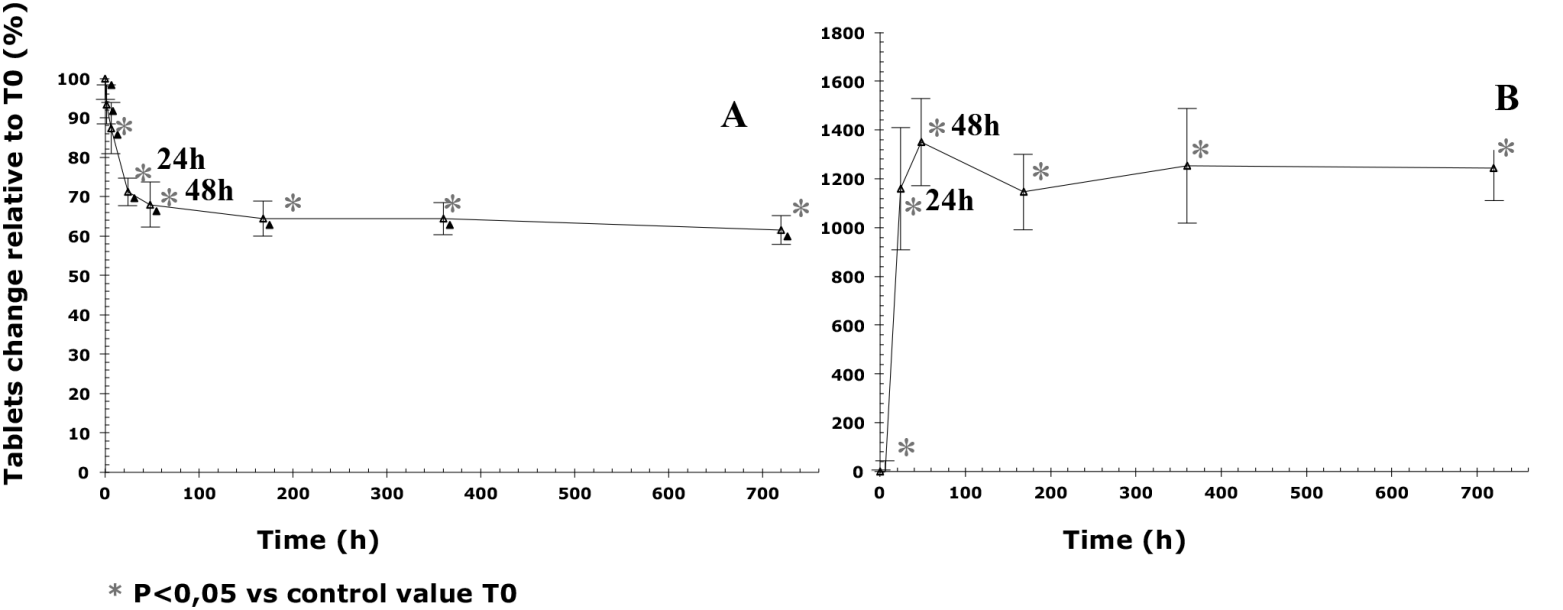
Changes over time in the hardness (A) and friability (B) of tablets stored at 25°C/60% RH in comparison with T0. The vertical bars represent the 95% confidence interval limits for the measured hardness (A) and friability (B) variation.

There was a significant decrease in tablets’ hardness (−32% relative to T0) during the first 2 days of storage. After 24 hours, the hardness decreased to 99.32 N, below the lower limit of the pre-specified range from European Pharmacopeia that allows for a 10% variation (Table 1, Fig. 5A). Friability increased 1300% between T0 and T48 from 0.036% to 0.44% (Table 1, Fig. 5B).

At 60% of relative humidity and 25°C, the penetration of water into the tablets was very fast and led to their softening.

### Changes in the misoprostol content in Cytotec tablets

**Figure 6:**
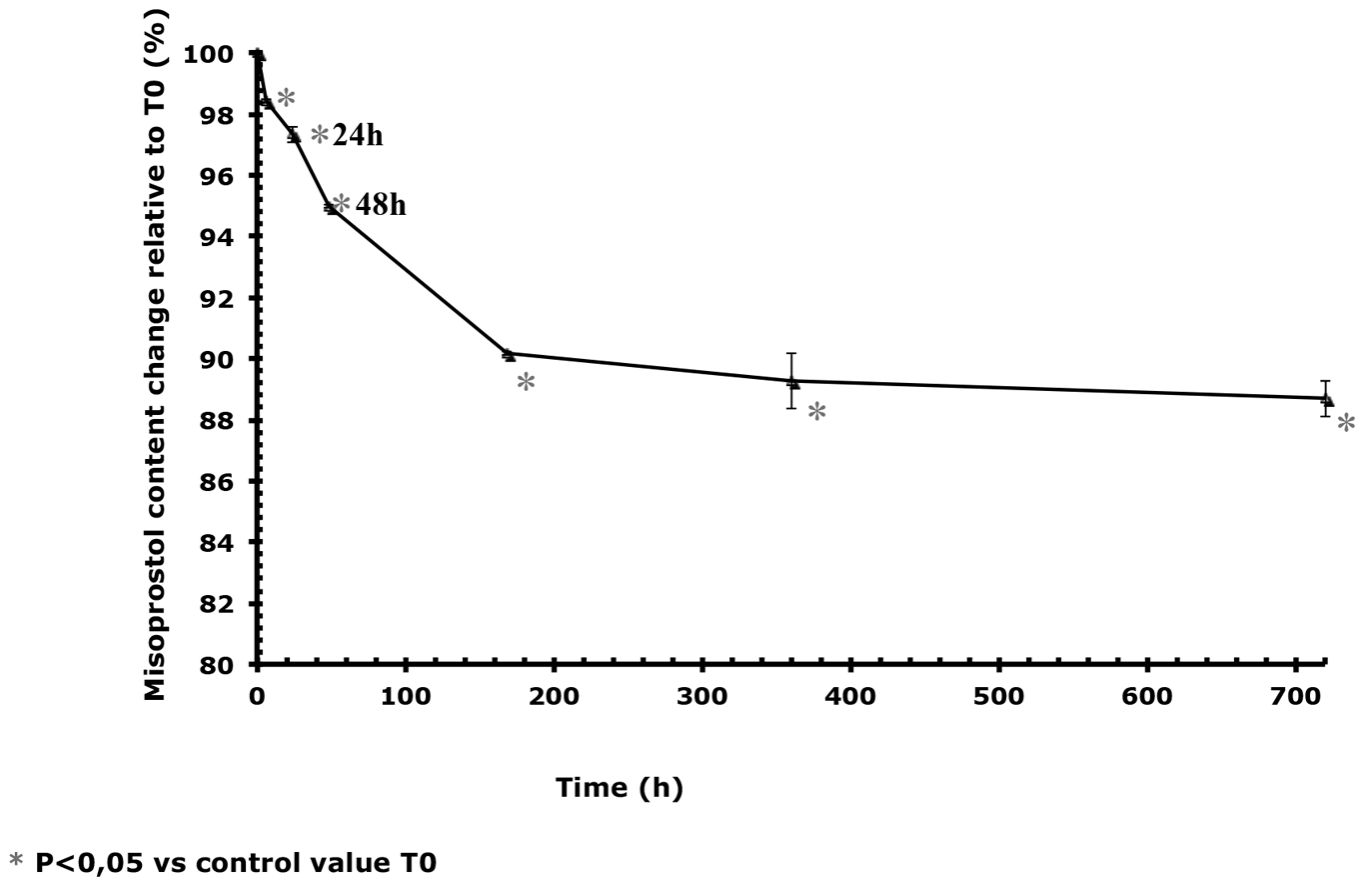
Changes over time in the misoprostol concentration of tablets stored at 25°C/60% RH in comparison with T0. The vertical bars represent the 95% confidence interval limits for the measured content variation.

The misoprostol content of the tablets decreased rapidly during the first week in environmental conditions (Table 1). The exposed tablets lost 5% of active ingredient by the first 48 hours and 10% by the end of one week (Fig. 6).

### Changes in misoprostol degradation products in Cytotec tablets

The transformation of misoprostol to inactive type A misoprostol appeared immediately after exposure to atmospheric conditions and the type A percentage per tablet was multiplied by 1.5 in less than 48 hours.

The percentage of type B was constant during the first 48 hours after exposure and gradually increased to almost double baseline levels, by one month after exposure. The 8-epi transformation by isomerisation of misoprostol did not begin immediately after storage. The percentage of 8-epi remained constant during the first 48 hours and then gradually increased, and doubled by 1 month of exposure.

## Discussion

This study showed that exposing Cytotec tablets to usual European humidity and room temperature, outside of their blister of protection, modifies the physical but more importantly the biological characteristics of the product.

Misoprostol, an E1-type prostaglandin, is known to be very unstable and must be stabilized in the form of a solid dispersion with Hydroxypropyl Methylcellulose (HPMC). The HPMC is designed to protect the misoprostol and to slow down the water penetration [26].

In our study, there was a large increase in misoprostol tablet water content 2 days after exposure to environmental conditions. This is important because this 2-day time period corresponds to a usual delay between mifepristone administration and misoprostol intake in most medical abortion regimens [27], [28].

All tests showed that the change in pharmaco-technical characteristics of tablets reached their maximum at 48 hours. This corresponds to the time required for the maximum penetration of water into the tablets when they are kept outside of the blister. The water content of the tablet is a pivotal parameter, as it initiates the degradation of misoprostol. The increase in the weight of tablets is the first sign of water penetration inside the tablets. Misoprostol tablets contain hydroxypropylmethylcellulose (HPMC) as an excipient, dedicated to protection of the active component, i.e. misoprostol, from ambient humidity. [26] The observed increase in tablets’ diameter and thickness shows that the atmospheric water penetrating into tablets makes the HPMC that they contain inflate. HPMC used in production of misoprostol may contain as much as 5% water. A drying process is included in the manufacturing process and all production rooms are monitored in terms of temperature and relative humidity (value below 37% at 18°C<T<25°C). This is why the specifications for misoprostol tablets total water content need to be limited to not more than 4.5% in order to avoid degradation of the active pharmaceutical ingredient. In our study, with water content reaching 5.53% at 24 hours, the protection of misoprostol by HPMC is widely exceeded. Kararli & Calatano [26] postulated that the stability of misoprostol was found to be at a maximum for an increase in water level below 2%, outside the intrinsic water content of the HPMC.

As the percentage of relative humidity increases, the HPMC becomes plasticized because of the increasing number of dissolved water molecules. This explains why the tablets become increasingly brittle which may result in a loss of compound at the time the patient takes it.

In preformulation studies, water was found to be one of the catalysts in the dehydration of compound. Therefore the role of water in the degradation of misoprostol is twofold, that of a plasticizer and a catalyst. [19], [26] As a consequence, it initiates the degradation of misoprostol in inactive substances.

The swelling of the HPMC and its plasticization allow water to access the molecules of misoprostol. When the amount of water is sufficient to swell the HPMC (48 hours), the degradation of misoprostol was initiated and went fast, reaching 5% at 48 hours and more than 10% after 7 days. The results show a loss of active misoprostol because it is transformed into 3 main inactive degradation products: type A and type B and 8-epimer misoprostol.

Stability studies of the misoprostol/HPMC dispersion have indicated that the first-order rate constants for misoprostol degradation increased as the water content of misoprostol increased. [19], [26], [29] Below two percent water content, the rate of misoprostol degradation was found to be minimal but in humid conditions with higher water content in the atmosphere, misoprostol is transformed by dehydration and isomerization in type A, type B and 8-epi misoprostol that are process impurities and are also degradation products. [29] Their levels are restricted in misoprostol active substance [30].

The water penetration, associated with a storage temperature of 25°C, speeds up the process of transforming the misoprostol into decomposition compounds. The only way to protect the misoprostol tablets from this adverse effect of water penetration is to store them in a sealed aluminium blister. Furthermore, it is important to note that these effects may be worse in countries with higher humidity levels.

In order to be able to deliver the adequate dosage to the patient, Cytotec blister packs have to be cut, which is frequently done several days prior to administration. This may result in tablets being stored outside the aluminium blister, or in an unsealed packaging. As this study shows, cutting the blister should be avoided due to the risk of damaging the packaging around tablets with consequential exposure of the tablets to environmental conditions.

In such conditions, at the time of drug intake, a decrease in active misoprostol components associated with an increase in inactive degradation products is expected to occur. Correct dosing is of crucial importance for clinical procedures that rely on misoprostol such as medical termination of pregnancy, medical management of miscarriage, and cervical ripening, because dosage recommendations for misoprostol in these indications are based on the lowest effective dose, with the intention to keep side effects at a minimum. Consequently, treatment guidelines do not contain a ‘safety margin’ to compensate for a reduced content of the active ingredient due to degradation. To our knowledge, no clinical studies were performed assessing the clinical impact associated with misoprostol degradation. And the clinical impact of a 10% loss of the active substance might be difficult to judge. Furthermore, dose-finding studies were done in incremental steps of 200 mcg as most tablets come in that size. In this situation we can derive the clinical impact of a reduced dose from the results of studies on cervical priming where a 50% reduction in dose (from 400 mcg to 200 mcg) let to a 75% reduction in successful dilatation (96.7% of cases compared to 23.3% of cases) [31].

Although there are no clinical studies to demonstrate reduced efficacy of misoprostol for its multiple clinical indications after exposure to atmospheric conditions, our results indicate that a reduction in clinical effectiveness is a real possibility. Further studies are needed to better understand the clinical impact in different indications and to develop strategies to avoid inadvertently opening of the blisters.

In the meantime pharmacists, physicians and patients should be reminded of the importance to store misoprostol tablets in the unopened aluminium blister until they are taken and to avoid any inadvertent opening when cutting the blister.

## Conclusions

The results of this study clearly show that there is a significant time-dependent decrease in Cytotec tablets technical-pharmaceutical characteristics when they are in contact with normal atmospheric conditions (as exist in Europe).

The 10% reduction in misoprostol content and the increase in degradation product content over 48 hours found in this study call for clinical studies focusing on associated clinical implication. There is a need for improving the drug packaging, and Health providers should be aware of the necessity of protecting the tablets in their original aluminium seal without damage.
